# Synthesis of Porous Polydimethylsiloxane Gold Nanoparticles Composites by a Single Step Laser Ablation Process

**DOI:** 10.3390/ijms222212155

**Published:** 2021-11-10

**Authors:** Mariapompea Cutroneo, Vladimir Havranek, Anna Mackova, Petr Malinsky, Letteria Silipigni, Petr Slepicka, Dominik Fajstavr, Lorenzo Torrisi

**Affiliations:** 1 Nuclear Physics Institute, AS CR, 250 68 Rez, Czech Republic; Havranek@ujf.cas.cz (V.H.); mackova@ujf.cas.cz (A.M.); malinsky@ujf.cas.cz (P.M.); 2 Department of Physics, Faculty of Science, University of J. E. Purkyně, České Mládeže 8, 400 96 Ústí nad Labem, Czech Republic; 3 Department of Physics (MIFT), Messina University, V.le F.S. D’Alcontres 31, 98166 Messina, Italy; lsilipigni@unime.it (L.S.); ltorrisi@unime.it (L.T.); 4 INFN, Sezione di Catania, Via S. Sofia 64, 95123 Catania, Italy; 5 Department of Solid State Engineering, Institute of Chemical Technology, 166 28 Prague, Czech Republic; petr.slepicka@vscht.cz (P.S.); dominik.fajstavr@vscht.cz (D.F.)

**Keywords:** laser ablation in medium, nanoparticles, polydimethylsiloxane, porous composite, curing time, scanning electron microscopy, absorbance

## Abstract

Typically, polymeric composites containing nanoparticles are realized by incorporating pre-made nanoparticles into a polymer matrix by using blending solvent or by the reduction of metal salt dispersed in the polymeric matrix. Generally, the production of pre-made Au NPs occurs in liquids with two-step processes: producing the gold nanoparticles first and then adding them to the liquid polymer. A reproducible method to synthetize Au nanoparticles (NPs) into polydimethylsiloxane (PDMS) without any external reducing or stabilizing agent is a challenge. In this paper, a single-step method is proposed to synthetize nanoparticles (NPs) and at the same time to realize reproducible porous and bulk composites using laser ablation in liquid. With this single-step process, the gold nanoparticles are therefore produced directly in the liquid polymer. The optical properties of the suspensions of AuNPs in distilled water and in the curing agent have been analyzed by the UV-VIS spectroscopy, employed in the transmission mode, and compared with those of the pure curing agent. The electrical dc conductivity of the porous PDMS/Au NPs nanocomposites has been evaluated by the I–V characteristics. Scanning electron microscopy (SEM) and energy dispersive X-ray (EDX) analysis have monitored the composition and morphology of the so-obtained composites and the size of the fabricated Au nanoparticles. Atomic force microscopy (AFM) has been used to determine the roughness of the bulk PDMS and its Au NP composites.

## 1. Introduction

Recently, elastomer composites are emerging as leading solutions for fabricating flexible and stretchable electrically conductive materials, promising candidates for the wearable electronics and sensors. 

Polydimethylsiloxane (PDMS) is an organosilicon polymer characterized by a low glass transition temperature T_g_, biocompatibility, good flexibility, good resistance to thermal and oxidative stability (over degradation), low cost, and chemical inertness. PDMS is being extensively investigated to develop biomedical micro or nano devices for biosensing platforms [1], cell culture substrates [2], and biomimetic devices [3]. However, PDMS exhibits difficulty of surface wetting with aqueous solvents because its surface is hydrophobic [4]. In PDMS, the CH_3_ groups can lead to the fixation of liquid molecules and gases as fluorescent dyes [5] and organic solvents [6]. To improve the wettability of PDMS, the oxygen plasma treatment has been used, and hydrophilic surfaces of PDMS [7] and hydrophilic patterns [8] have been created using ion lithography. These approaches do not ensure the durability and stability of coatings [9] and have shown some limitations and temporary hydrophilicity of PDMS [10]. The limitations of weak interfacial bonding and low robustness [11] can be overcome by blending it with another polymer or incorporating fibres and nanoparticles into PDMS [12]. Au-based nanoparticles, nanostructures, and nanoclusters [13,14] have recently been receiving a lot of attention in fundamental physics [15], nanotechnology, and biomedicine [13]. In particular, Au nanoclusters (Au NCs) have demonstrated stability, reduced toxicity, and strong fluorescent emission, which permits the quantification of nucleus-targeting molecules with single-particle sensitivity [16,17]. The inclusion of NPs in PDMS makes possible the development of contactless biosensors because the embedded NPs work as localized sensors for the conversion of chemical or biomolecular information into a signal that can be physically readable [16,17]. AuNPs can be easily modified [18] with biomolecules such as DNAs and proteins by thiol and amine via Au-S or Au-N bonds without destroying the activity of biomolecules. 

Different size- and shape-dependencies of particles affect their optical properties, making them useful for surface-enhanced Raman scattering (SERS) or the so-called plasmonic sensing [19].

Several attempts have been made to synthesize nanoparticles embedded in the PDMS matrix: mixing pre-made nanoparticles into the polymer matrix, using blending solvents, reduction of metal salt, physical and chemical vapor deposition, and ion implantation. 

Several studies [20] confirm that the critical parameter for laser ablation in medium, more than laser fluence, wavelength, and pulse duration, is environment. It is well-known that the ablation rate decreases with increasing medium density and boiling point [21]. It also decreases with increasing level of liquid medium, which should be crossed by the laser beam before it hits the solid target, leading to a reduction of the number of produced nanoparticles. 

This study describes the attempt of producing Au NPs by means of laser ablation in the organic polymeric solvent, embedding in this way the NPs in the PDMS polymeric matrix in one step, avoiding the use of pre-formed NPs and bypassing external reducing or stabilizing agents.

## 2. Results

**Figure 1:**
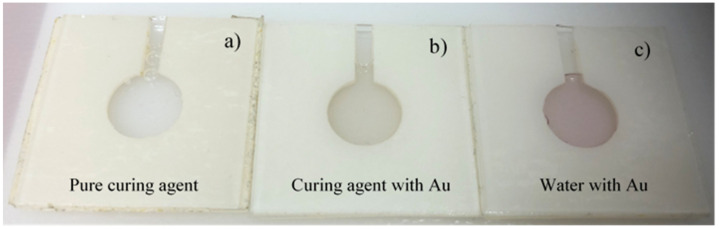
Photos of the homemade plastic holders containing the pure curing agent (**a**), the suspension of curing agent with AuNPs (**b**), and the suspension of water with AuNPs (**c**).

To synthetize the AuNPs by laser ablation, water and curing agent were individually used as liquid. After the laser irradiation in the distilled water containing the gold target, the water turned from transparent to ruby red due to the presence of Au NPs (suspension 1). When the curing agent is laser irradiated, it turns from transparent to yellow because now it contains the Au nanostructures (suspension 2), as one can see in the circular containers shown in Figure 1.

### 2.1. Optical Absorbance Measurement

Surface plasmon resonance (SPR) is the collective oscillation of electrons on the surface of metal (gold in our case) excited by photons, which results in a peak observed in the absorption spectrum of a sample. The position and width of the SPR absorption peak depends on both the size and agglomeration of the gold NPs. 

**Figure 2:**
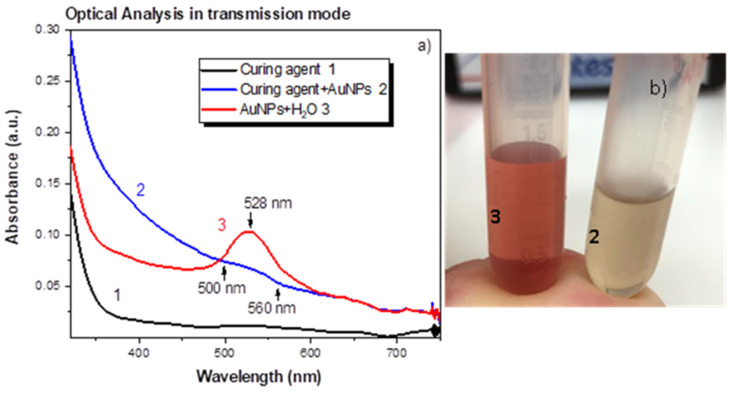
UV-Visible spectra of the pure curing agent (black curve 1), curing agent with AuNPs (blue curve 2) water with AuNPs (red curve 3) suspensions (**a**), a picture of curing agent with AuNPs (2), and water with AuNPs (3) suspensions (**b**).

As one can see in Figure 2—red curve 3, the Au NPs in water have an absorbance peak, given in arbitrary units (a. u.), centered at about 528 nm due to the Au NPs SPR, confirming the formation of a spherical gold nanoparticle with a size of about 20 nm [22]. A visible background is also present, due to scattering. With regard to the intensity of the SPR peak [23], it is related to the concentration of gold NPs. Considering that the gold NPs were spherical in shape and their density was the same as bulk gold (i.e., 19.3 g/cm^3^), the concentration of gold NPs has been calculated by weighing the lasered solid gold target before and after the laser ablation and is about 1.1 × 10^13^ NPs/cm^3^. The uncertainty in the NPs concentration is of about 5%.

**Figure 3:**
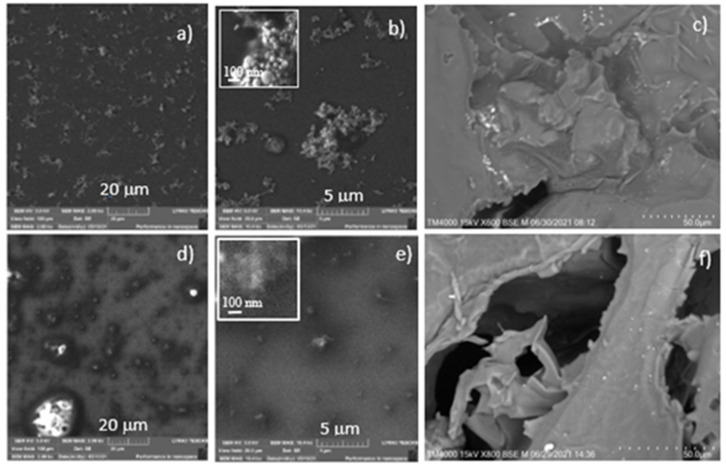
SEM images with different magnifications of Si substrates covered with distilled water +AuNPs (**a**,**b**) and curing agent +AuNPs (**d**,**e**); 600× and 800× magnified SEM images of porous PDMS embedded with E_AuNPs (**c**) and C_AuNPs (**f**) are shown. In the inserts of (**b**,**e**), the agglomerated AuNP structures are displayed.

The suspension of the curing agent containing the AuNPs shows higher absorbance due to the presence of bigger-in-size AuNP nanostructures (as can be seen in Figure 3c) and of carbon groups generated during the laser irradiation as shown in Figure 2—blue curve 2. It also shows a poorly resolved SPR peak in the (500–560) nm region, likely because of the very low concentration of gold NPs. A background, due to scattering, is also visible. The lowest Au NPs concentration is also observed by the yellowish colour shown by the suspension 2 (see Figure 2b). The uncertainness on the absorbance evaluation is about 6%.

### 2.2. Electrical Response of Porous Composites

The electrical *dc* conductivity parameter, σ, given in (Ωx cm)^−^^1^ was calculated from the acquisition of the I–V characteristics for the porous samples. These latter samples were prepared in the form of a cube with length of 8.0 mm, width of 8.0 mm, and thickness of 7.0 mm. The measurement of electrical conductivity σ was performed by placing the sample between two copper electrodes with a surface a little larger than the cube face area. The electrical conductivity σ for the *bulk* PDMS is very low, about 2.5 × 10^−16^ (Ω·cm)^−^^1^, as reported in literature [24]. The values of the electrical *dc* conductivity calculated for the porous C_AuNPs and E_AuNPs PDMS composites were 1.8 × 10^−7^ (Ω·cm)^−1^ and 1.09 × 10^−7^ (Ω·cm)^−1^, respectively. The electrical conductivity for a bulk of gold is 5.96 × 10^5^ (Ω·cm)^−1^ [25]. The uncertainty in the electrical conductivity is of about 5%.

### 2.3. Contact Angle Measurement

**Figure 4:**
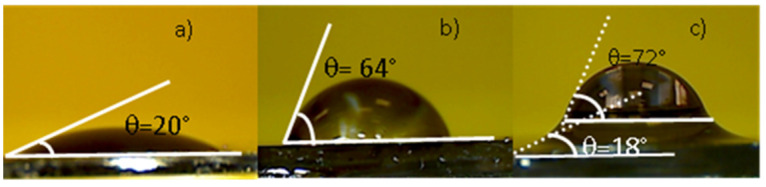
Photos of the contact angle measured on silicon substrates covered with: distilled water (**a**), a suspension of distilled water with AuNPs (**b**) and a suspension of curing agent with AuNPs (**c**).

The wettability of surfaces revealed through the measure of the contact angle (θ), given in degree angle, resulting from the forces applied to the solid-gas, solid-liquid, and liquid-gas interfaces is shown in Figure 4. Silicon substrates were at first covered with a few drops of distilled water, distilled water with AuNPs, and curing agent + AuNPs and then dried to be ready to measure the contact angle of the drop profile. The contact angle in silicon substrate with distilled water is about 20° (see Figure 4a), indicating a strong hydrophilic nature. In the Si substrate with AuNPs + H_2_O, the higher inhomogeneity of the liquid medium decreases the surface energy of the substrate material. 

This reduces the wettability of the molecular liquid as indicated by an increased contact angle of 64° (see Figure 4b). AuNPs on the Si surface enhance the adhesion forces. In Figure 4c, the contact angle measurement on the silicon substrate covered with curing + AuNPs, due to the complex nature of the liquid, displays two contact angles referred to as the formed layers. The wetting angle is measured with accuracy of about 7%.

### 2.4. Morphological Characterization by SEM and AFM Analyses

Evaluation of the morphology of the AuNPs produced by the laser ablation was performed by the SEM images analysis on different regions of the silicon substrate coated with the prepared suspensions. Figure 3 shows the 2.08 kX (a, d), 10.4 kX (b, e) magnified SEM images of Au NPs distributed on the surface of Si substrates. 

The SEM images in the inserts are 104 kX magnified, while the ones of porous PDMS composites are 600× and 800× magnified. The AuNPs average size is about ~20 nm in water (a,b) and hundreds of nm in the curing agent. The NPs have a size distribution with a deviation of about 10%. In both cases, agglomerated AuNPs structures are evident, as displayed in the inserts of Figure 3b,e, respectively. The bright points emerging in Figure 3c,f indicate the AuNP structures in the porous PDMS E_AuNPs and C_AuNPs composites, showing a higher concentration in E_AuNPs.

**Figure 5:**
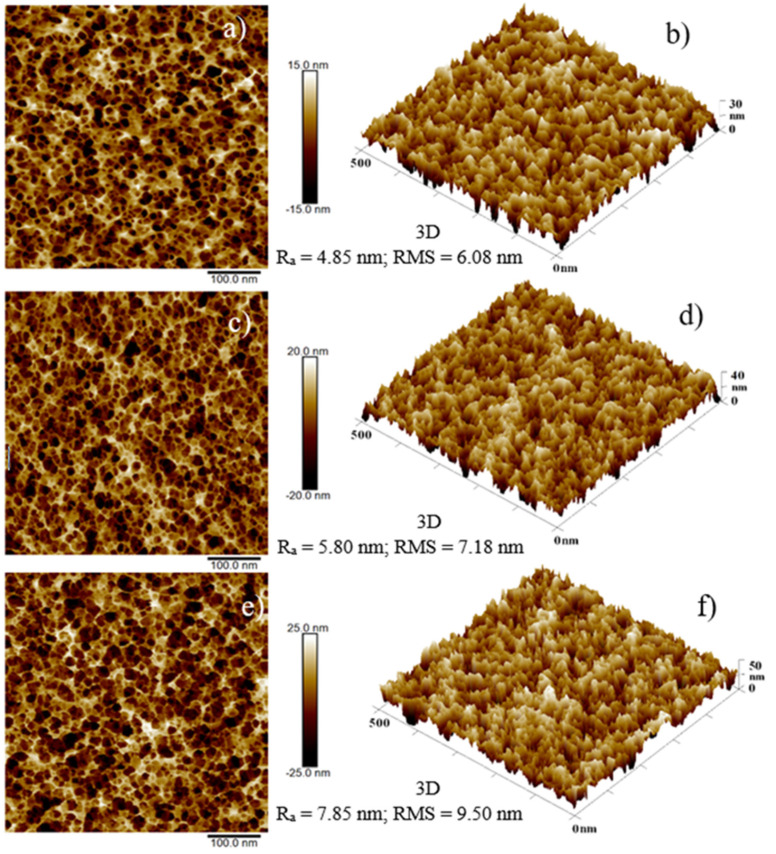
2D AFM images and corresponding 3D AFM images of virgin *bulk* PDMS (**a**,**b**), *bulk* PDMS E_AuNPs composite (**c**,**d**) and *bulk* PDMS C_AuNPs composite (**e**,**f**).

Figure 5 shows 2D (a, c, e) and 3D AFM (b, d, f) images of virgin *bulk* PDMS (a), *bulk* PDMS E_AuNPs (b), and *bulk* PDMS C_AuNPs (c).

The differences between the surface (basic) of the investigated area and the rough surface (real) in the investigated area are 130%, 192%, and 259%, respectively, pointing out an increase in roughness in agreement with the reported RMS: 6.08, 7.18, and 9.50 nm. The average roughness of 4.85, 5.80, and 7.85 nm is evaluated for the three investigated samples, respectively. The roughness measurements are affected by an uncertainty of about 10%.

In Table 1 is listed the composition of the investigated suspensions obtained by EDX analyzing the emission yield of the characteristic X-ray lines. The atomic percentage values listed in table confirm the presence of a lower concentration (0.07 atom %) of AuNPs in the suspension containing the curing agent with respect to that in distilled water. An enhancing of the carbon and oxygen content, up to 58% and 70.1%, respectively, is also observed between the two suspensions of Au NPs.

The observed enhancement of the carbon content in the suspension of curing agent +AuNPs is probably due to the carbonization induced by the laser irradiation This last observation could cause the oxidation of the Si-H bond to Si-O-Si, thus justifying the detected increase in O. The EDX composition is given with an error of about 6%.

## 3. Discussion

The laser ablation in liquid can include different approaches, where NPs can be produced irradiating a solid target, a molecular precursor, or both together. Typically, when the laser is focused on the surface of the solid target, placed in the liquid medium, a plasma plume is generated at the liquid-solid interface. The plume develops a bubble of hot compressed gas, which expands at high velocity. The expansion velocity increases with the temperature and decreases with the liquid viscosity. The enhancement of the temperature and pressure in the plume occurs due to collisions between ions, atoms, and molecules. The confinement by liquid results in the cooldown of the plume, generation of new solid phase structures, atom aggregation that forms nanoparticles ascribed to crystallization effects, molecular scissions, and grain growth. 

The size and shape of produced NPs have been investigated by optical spectrometry. The optical spectrum observed in Figure 2 shows the SPR band around 528 nm corresponding to spherical size of about 20 nm. The SPR band is affected by the size of the NPs, and a slight red shift is observed in Suspension 2, confirming the increased Au structure size. The broader band in curing agent +AuNPs indicates wider size distribution. The lower SPR band intensity in Suspension 2 than in Suspension 1 can be explained by a lower AuNPs concentration. This minor AuNPs amount is due to the lowest plasma plume expansion velocity in this medium having higher viscosity than distilled water according to SEM and EDX analyses. 

To assess the morphology and dispersion of sintered NPs, wettability measurements have been performed. Many factors can affect the wettability from the NPs dimension to the roughness of the substrate. The surface roughness represents a crucial parameter concerning the wettability. The contact angle decreases with the roughness decrease and correspondingly to the surface wettability increase. Based on the observation of the liquid droplet profile reported in Figure 4, the AuNPs in water with an average dimension of 20 nm, lower than in the curing agent, enhance the contact angle, reducing the wettability. The contact angle observed for curing agent+ AuNPs consists in two contact angles. The former, specified by the bottom bold/dotted white lines at 18°, indicates high hydrophilicity with the silicon surface, probably due to the presence of carbon fragments and Au nanostructures, which adhere strongly, with silicon decreasing the contact angle. The latter, indicated by the white bold/dotted lines, at 72° suggests still high hydrophilicity, probably due to the presence of silicate in the lighter layers that do not contain rough structures. The wettability results have indicated surface roughness changes consistent with SEM and AFM analyses. 

AFM analysis has shown an increased average roughness, due to the presence of hosted structures in the composites, going from virgin *bulk* PDMS to *bulk* PDMS E_AuNPs to *bulk* PDMS C_ AuNPs, suggesting that the higher viscosity of the liquid polymer, with respect to the water, can be responsible for the larger AuNPs formations. The slightly higher value of electrical conductivity obtained in the C_AuNPs PDMS composite could be due more to the carbonization of the suspension than the presence of AuNPs, unlike in the E_AuNPs PDMS composite, as indicated by the increase of carbon concentration in curing agent+ AuNPs according to EDX analysis.

## 4. Materials and Methods

The PDMS kit containing the elastomer and the curing agent was purchased from Sylgard 184 (Dow Corning). The curing agent is composed of dimethyl, methylhydrogen siloxane, dimethyl siloxane, dimethylvinylated, and trimethylated silica, tetramethyl-tetravinyl cyclotetrasiloxane, and ethyl benzene [26]. 

A Litron Q-switching Nd:YAG laser [27] operating at the 1064 nm wavelength, with a 5 ns pulse duration, 200 mJ pulse energy, 1 mm^2^ focused spot, and 10 Hz pulse repetition rate, was employed. A gold solid target purchased by Goodfellow with a surface of 1 cm^2^ and 1 mm thickness was placed on the bottom of a glass beaker filled once with 8 mL of distilled water (suspension 1) and again with 8 mL of curing agent (suspension 2). The laser beam impinges against a prism at a 126 cm distance; then, it is focused by a lens 9 cm from the prism and 50 cm from the solid target immersed in the liquid. The laser was focused on the metallic target with a 50 cm focal length convergent lens to obtain a 1 mm spot diameter. The laser irradiation lasted 5 min. 

The concentration of AuNPs evaluated by weighing the gold target before and after 5 min of laser ablation is about 0.9 mg/mL in both the distilled water and the curing agent. Suspension 1, consisting of distilled water, with a density of 1 g/cm^3^ and a viscosity of 1 centipoise (cP), and AuNPs, was mixed into the elastomer and native curing agent in a 10:1 weight ratio and was named E_AuNPs. Suspension 2, consisting of the curing agent, with a density of 0.965 gr/cm^3^ and viscosity of 14 cP, and AuNPs, was mixed into an elastomer in a weight ratio of 10:1 and was named C_AuNPs. 

**Figure 6:**
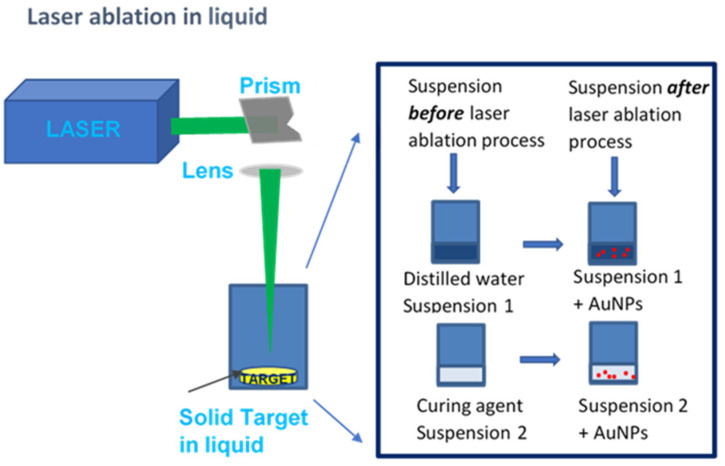
Sketch of the set-up used for the synthesis of Au NPs by the laser ablation in medium.

Subsequently, 1.1 g from both E_AuNPs and C_AuNPs solutions was used to prepare porous PDMS_AuNPs composites, whereas 0.9 g from both E_AuNPs and C_AuNPs was used to prepare *bulk* PDMS_AuNPs composites. A sketch of the experimental set-up is shown in Figure 6. 

The 1 mm thick *bulk* PDMS AuNPs composites were realized by pouring the suspensions into two Petri dishes, which were placed in an oven at 100 °C for 45 min and then peeled off from the Petri dishes. 

**Figure 7:**
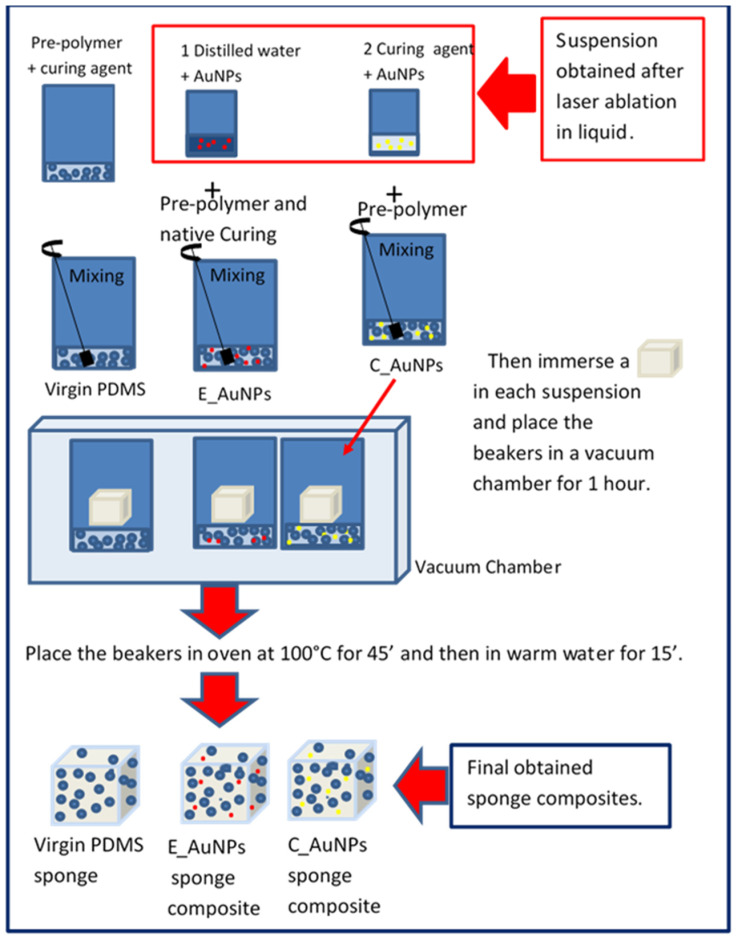
Sketch of the process used for the preparation of virgin PDMS, E_Au NPs, and C_AuNPs PDMS sponge composites.

The preparation of the porous PDMS AuNPs composite, based on the sugar template method [28], is shown in Figure 7.

Two sugar bricks were immersed in two beakers containing 1.1 g of E_AuNPs and C_AuNPs solutions, respectively. Successively degassing in a vacuum chamber for 1 h assisted the capillary infiltration of suspensions into the voids of the sugar bricks. The obtained sugar bricks filled by the two suspensions were cured in an oven for 45 min at 100 °C and then they were leached out by rinsing in distilled water at 60 °C for 15 min. The final PDMS_AuNPs sponges obtained from E_AuNPs and C_AuNPs were dried at room temperature for 24 h. 

The PDMS AuNPs sponge composites synthesized revealed a common porous interconnected structure, a common size of 8 × 8 × 7 mm, but a different colour. The *bulk* and the porous E_AuNPs composites had a red ruby appearance, while the *bulk* and the porous C_AuNPs composites had a light-yellow look, as shown in Figure 7.

To evaluate the AuNP size, the UV-Vis spectroscopy, was employed. An AvaSpec-2048 spectrometer with an UB-600 lines/mm grating and a bandwidth from 195 to 757 nm was used in the (350–750) nm wavelength range. Halogen/deuterium lamps were used as sources for the optical analysis in the transmission mode. An optical fibre connected to the source illuminated at 0° the front side of the holder containing the suspension, whereas another optical fibre was placed behind the holder at −180°. The transmittance spectra of the native curing agent, the curing agent containing AuNPs, and the water containing AuNPs were measured using a homemade plastic holder [29] and converted into absorbance spectra. 

The scanning electron microscopy (SEM) TESCAN LYRA3 GMU (Tescan, CZ) microscope monitored the surface morphology of the *bulk* samples in the secondary electron mode using an acceleration voltage of 5 kV. The view fields were of about 100 and 20 and 2 μm. The porous composites were investigated in the backscattered electrons (BSE) mode at 15 kV and at a view field of 200 μm. Samples were coated with a layer of Pt with thickness less than 50 μm to minimize charging effects and consequently to improve the contrast and the image quality.

Using the SEM microprobe, energy dispersive X-ray (EDX) analysis was performed to determine the surface elemental ccomposition of the investigated samples by detecting the characteristic X-ray of the elements present on their surfaces. The relative spectra were obtained using a 5 keV electron beam with a beam current of 60 pA and a 50 μs dwell time per pixel, spot size 60 nm.

The surface roughness and the structures size of *bulk* composites were studied in air and at room temperature by the atomic force microscopy (AFM) images [30] acquired by a Dimension ICON (Bruker Corp., Billerica, MA, USA) microscope, in quantitative nanoscale mechanical (QNM) mode. A silicon tip on Nitride Lever SCANASYST-AIR with spring constant 0.4 N/m was used. AFM images were taken with an exposure of 1 s. The data were processed employing the NanoScope Analysis software [28]. The analyzed area was of about 500 nm in 2D and 3D images. Surface average roughness (R_a_) and the mean roughness parameter (RMS) were calculated.

The modification of the wettability exhibited by the PDMS_AuNP composites was investigated by the sessile drop method using an optical microscope [31]. A drop of 3 μL of the prepared suspension was poured using a micrometric syringe on the sample surface to measure the contact angle. Five contact angles in different areas of the same sample were measured, and the obtained average value was evaluated. For each sample, the reported results were obtained as the average on three droplets, and the overall accuracy in the measurements was better than ±5°.

The electrical response of the porous samples was monitored by a Keithley 6517B electrometer suited for measurement of resistance up to 10^18^ Ω, voltages from 1 μV up to 200 V and with an input impedance exceeding 200 TΩ. Two rectangular copper electrodes were placed in parallel on the sponge surface with a separation distance of 7 mm. The current–voltage (I–V) characteristics of the virgin porous PDMS and the PDMS composites containing AuNPs were determined automatically by applying voltages ranging between −100 and +100 V and measuring the relative current.

## 5. Conclusions

In summary, distilled water and curing agent have been used as media to produce Au nanoparticles by the laser ablation technique in liquid starting from a solid target of Au. With this method, the gold nanoparticles have been synthesized and simultaneously embedded in *bulk* and porous PDMS in a single-step obtaining PDMS_AuNPs composites without the use of additional reducing agents or further manipulations. 

The suspension of distilled water and AuNPs is ruby red due to the presence of spherical gold nanoparticles with a size of about 20 nm, while the curing agent containing gold nanoparticles is yellow because of the presence of larger distributions of gold nanostructures as shown in the SEM images. These larger Au nanostructures can be due to the lower expansion velocity of the plasma plume in the high viscosity liquid. 

The presence of AuNPs in both suspensions is confirmed by the optical spectra that show a more or less pronounced SPR peak in the (500–560) nm range.

Even if characterized by a minor AuNP concentration, the single-step preparation presented in this paper is innovative and useful for preparing polymers containing metallic NPs with direct synthesis by laser without risks of liquid contaminations. Through the “correction” of the viscosity of the curing agent, the single-step method could also open the way to the tailoring of the size and concentration of prepared nanoparticles.

## Figures and Tables

**Table 1:** The elemental composition in atomic percentage of the suspensions of AuNPs in distilled water and in curing agent, deposited on Si.

SAMPLE	Atomic %			
	C	O	Si	Au
Suspension of AuNPs in distilled water drop-casted on a Si substrate	14.02	4.17	80.30	1.51
Suspension of curing agent +AuNPs drop-casted on a Si substrate	33.35	14.21	52.37	0.07

## References

[1] SadAbadi H., Badilescu S., Packirisamy M., Wüthrich R. (2013). Integration of gold nanoparticles in PDMS microfluidics for lab-on-a-chip plasmonic biosensing of growth hormones. Biosens. Bioelectron..

[2] Bai H.-J., Gou H.-L., Xu J.-J., Chen H.-Y. (2010). Molding a Silver Nanoparticle Template on Polydimethylsiloxane to Efficiently Capture Mammalian Cells. Langmuir.

[3] Bhatia S.N., Ingber D.E. (2014). Microfluidic organs-on-chips. Nat. Biotechnol..

[4] Vlachopoulou M.-E., Petrou P., Kakabakos S., Tserepi A., Beltsios K., Gogolides E. (2009). Effect of surface nanostructuring of PDMS on wetting properties, hydrophobic recovery and protein adsorption. Microelectron. Eng..

[5] Abate A.R., Lee D., Do T., Holtze C., Weitz D.A. (2008). Glass coating for PDMS microfluidic channels by sol–gel methods. Lab Chip.

[6] Lee J.N., Park A.C., Whitesides G.M. (2003). Solvent Compatibility of Poly(dimethylsiloxane)-Based Microfluidic Devices. Anal. Chem..

[7] Mata A., Fleischman A.J., Roy S. (2005). Characterization of Polydimethylsiloxane (PDMS) Properties for Biomedical Micro/Nanosystems. Biomed. Microdevices.

[8] Cutroneo M., Havranek V., Mackova A., Malinsky P., Torrisi A., Silipigni L., Sofer Z., Torrisi L. (2020). Selective modification of electrical insulator material by ion micro beam for the fabrication of circuit elements. Radiat. Eff. Defects Solids.

[9] Bodas D., Rauch J.-Y., Khan-Malek C. (2008). Surface modification and aging studies of addition-curing silicone rubbers by oxygen plasma. Eur. Polym. J..

[10] de Menezes C.A. (2010). Highly stable hydrophilic surfaces of PDMS thin layer obtained by UV radiation and oxygen plasma treatments. Phys. Status Solidi C.

[11] Yin J., Yang Y., Hu Z., Deng B. (2013). Attachment of silver nanoparticles (AgNPs) onto thin-film composite (TFC) membranes through covalent bonding to reduce membrane biofouling. J. Membr. Sci..

[12] Ibrahim I.D., Jamiru T., Sadiku R., Kupolati W.K., Agwuncha S.C. (2016). Impact of Surface Modification and Nanoparticle on Sisal Fiber Reinforced Polypropylene Nanocomposites. J. Nanotechnol..

[13] Torrisi L., Silipigni L., Restuccia N., Cuzzocrea S., Cutroneo M., Barreca F., Fazio B., Di Marco G., Guglielmino S. (2018). Laser-generated bismuth nanoparticles for applications in imaging and radiotherapy. J. Phys. Chem. Solids.

[14] Bai L., Zhu L., Ang C.Y., Li X., Wu S., Zeng Y., Ågren H., Zhao Y. (2014). Iron(III)-Quantity-Dependent Aggregation-Dispersion Conversion of Functionalized Gold Nanoparticles. Chem.-A Eur. J..

[15] Torrisi L., Cutroneo M., Ceccio G. (2014). Effect of metallic nanoparticles in thin foils for laser ion acceleration. Phys. Scr..

[16] Chevrier D.M., Chatt A., Zhang P. (2012). Properties and applications of protein-stabilized fluorescent gold nanoclusters: Short review. J. Nanophotonics.

[17] Wang Y., Chen J., Irudayaraj J. (2011). Nuclear Targeting Dynamics of Gold Nanoclusters for Enhanced Therapy of HER2+ Breast Cancer. ACS Nano.

[18] Johnson J.A., Dehankar A., Robbins A., Kabtiyal P., Jergens E., Lee K.H., Johnston-Halperin E., Poirier M., Castro C.E., Winter J.O. (2019). The path towards functional nanoparticle-DNA origami composites. Mater. Sci. Eng. R.

[19] Torrisi L., Restuccia N., Cuzzocrea S., Paterniti I., Ielo I., Pergolizzi S., Cutroneo M., Kovacik L. (2017). Laser-produced Au nanoparticles as X-ray contrast agents for diagnostic imaging. Gold Bull..

[20] Patel D., Singh R.P., Thareja R.K. (2014). Craters and nanostructures with laser ablation of metal/metal alloy in air and liquid. Appl. Surf. Sci..

[21] Hamad A., Li L., Liu Z., Zhong X.L., Wang T. (2015). Picosecond laser generation of Ag–TiO_2_ nanoparticles with reduced energy gap by ablation in ice water and their antibacterial activities. Appl. Phys. A.

[22] Kodeary A.K., Gatea M.A., Haddawi S.F., Hamidi S.M. (2020). Tunable thermo piezo plasmonic efect on core/shell nanoparticles under laser irradiation and external electric feld. Opt. Quantum Electron..

[23] Huang X., El-Sayed M.A. (2010). Gold nanoparticles: Optical properties and implementations in cancer diagnosis and photothermal therapy. J. Adv. Res..

[24] http://www.mit.edu/~6.777/matprops/pdms.htm.

[25] https://www.Thoughtco.com/table-of-electrical-resistivity-conductivity-608499.

[26] Goyal A., Kumar A., Patra P.K., Mahendra S., Tabatabaei S., Alvarez P.J.J., John G., Ajayan P.M. (2009). In situ Synthesis of Metal Nanoparticle Embedded Free Standing Multifunctional PDMS Films. Macromol. Rapid Commun..

[27] https://litron.co.uk/2021.

[28] Cutroneo M., Havranek V., Semian V., Torrisi A., Mackova A., Malinsky P., Silipigni L., Slepicka P., Fajstavr D. (2021). Porous polydimethylsiloxane filled with graphene-based material for biomedicine. J. Porous Mater..

[29] Torrisi L., Cutroneo M., Silipigni L., Barreca F., Fazio B., Restuccia N., Kovacik L. (2018). Gold nanoparticles produced by laser ablation in water and in graphene oxide suspension. Philos. Mag..

[30] http://nanoscaleworld.bruker-axs.com/nanoscaleworld/forums/t/812.aspx.

[31] Cutroneo M., Mackova A., Torrisi L., Lavrentiev V. (2017). Laser ion implantation of Ge in SiO_2_ using a post-ion acceleration system. Laser Part. Beams.

